# Validation of pooled genotyping on the Affymetrix 500 k and SNP6.0 genotyping platforms using the polynomial-based probe-specific correction

**DOI:** 10.1186/1471-2156-10-82

**Published:** 2009-12-14

**Authors:** Ramani Anantharaman, Fook Tim Chew

**Affiliations:** 1 Department of Biological Sciences, National University of Singapore, Science Drive 4, Singapore 117543

## Abstract

**Background:**

The use of pooled DNA on SNP microarrays (SNP-MaP) has been shown to be a cost effective and rapid manner to perform whole-genome association evaluations. While the accuracy of SNP-MaP was extensively evaluated on the early Affymetrix 10 k and 100 k platforms, there have not been as many similarly comprehensive studies on more recent platforms. In the present study, we used the data generated from the full Affymetrix 500 k SNP set together with the polynomial-based probe-specific correction (PPC) to derive allele frequency estimates. These estimates were compared to genotyping results of the same individuals on the same platform, as the basis to evaluate the reliability and accuracy of pooled genotyping on these high-throughput platforms. We subsequently extended this comparison to the new SNP6.0 platform capable of genotyping 1.8 million genetic variants.

**Results:**

We showed that pooled genotyping on the 500 k platform performed as well as those previously shown on the relatively lower throughput 10 k and 100 k array sets, with high levels of accuracy (correlation coefficient: 0.988) and low median error (0.036) in allele frequency estimates. Similar results were also obtained from the SNP6.0 array set. A novel pooling strategy of overlapping sub-pools was attempted and comparison of estimated allele frequencies showed this strategy to be as reliable as replicate pools. The importance of an appropriate reference genotyping data set for the application of the PPC algorithm was also evaluated; reference samples with similar ethnic background to the pooled samples were found to improve estimation of allele frequencies.

**Conclusion:**

We conclude that use of the PPC algorithm to estimate allele frequencies obtained from pooled genotyping on the high throughput 500 k and SNP6.0 platforms is highly accurate and reproducible especially when a suitable reference sample set is used to estimate the beta values for PPC.

## Background

Genome-wide SNP association screening has become a launching pad to the identification of genes or loci contributing to the susceptibility of complex diseases. With the advent of high-throughput genotyping microarrays such as those from Affymetrix and Illumina, a genome-wide scan for up to half a million genetic variants has become possible for even smaller laboratories which normally wouldn't be able to afford the manpower for large scale genotyping. In either platforms, single nucleotide polymorphisms (SNPs) were chosen as the markers of choice due to their abundance in the genome, their bi-allelic nature, and because they are stably inherited from generation to generation [1]. When studying complex human diseases with an apparent genetic basis, genome-wide scans used in the context of case-control association studies have shown some success in identifying multiple genes of small effect size that are likely to influence the various quantitative traits observed in these diseases [2]. Association of SNPs to a phenotype is usually identified by differences in allele frequencies of the variant between case and control samples. While other factors such as population stratification, epistasis, pleiotropy and gene-environment interactions may play a part in the phenotypic expression of differently observed allele frequencies, casting a genome-wide net to "fish" for susceptibility genes allows researchers to perform an un-biased initial round of screening to obtain a list of leads for more focused analysis in follow-up studies. Such an approach, known as the two-stage study design, has been shown to improve statistical power and reduce measurement errors [3].

For a genome-wide case-control study to be able to associate a particular genetic variant present at a frequency of at least 5% in the population with about 80% power, the samples that would have to be genotyped would number in the thousands [4]. Despite the ready availability of high throughput genotyping microarrays and the low cost per genotype, the cost of individually genotyping thousands of samples remain prohibitive outside of large-scale consortia. To overcome the limitations of cost, time and labor associated with large-scale individual genotyping, genotyping of pooled samples or the combination of SNP Microarrays and DNA Pooling (SNP-MaP) has been utilized [5]. The benefits of SNP-MaP were easy to appreciate. In principle, the allele frequencies of 1000 samples could be measured from one or a few pooled samples, rather than from 1000 individual samples, which represented an increase in efficiency of at-least a few hundred-fold. SNP-MaP has hence been used extensively in association studies of complex human diseases such as schizophrenia [6,7], rheumatoid arthritis [8], mild mental impairment [9], bipolar disorder. [10], etc. Numerous other studies have also been carried out just testing the viability and accuracy of SNP-MaP [5,11-19].

The accuracy and validity of pooled genotyping on microarrays has been extensively studied on the Affymetrix 10 k [9,11-15] and 100 k SNP array sets [16]. The recent success of genome-wide association studies using the Affymetrix 500 k array set has given credence to the usage of this platform in individual genotyping [20]. However, the accuracy of this platform in allelotyping pooled DNA samples has yet to be extensively evaluated. While pooled genotyping on the 500 k array set has indeed been performed [5,19], only Docherty et al. evaluated both the chips available in the array set. While the 500 k array set had been available for two years (2005-2007) and subsequently replaced by the even-higher throughput SNP5.0 and SNP6.0 arrays, only two publications on pooled genotyping using these higher throughput platforms have been released. This dearth of work could possibly be due to some apprehension about the performance of these higher throughput arrays due to the reduction in the number of probes per SNP, reducing available information thus potentially affecting the accuracy of genotype calls [15].

The purpose of this study is to comprehensively evaluate the ability of these higher throughput SNP genotyping platforms to estimate allele frequencies from pooled DNA samples. The full repertoire of 500,568 SNPs from the Affymetrix 500 k array set is used as the basis of an un-biased evaluation of the accuracy of pooled genotyping in comparison to allele frequencies obtained from individual genotyping with estimated allele frequencies calculated using the PPC algorithm [21]. This comparison is extended to the latest genotyping array from Affymetrix, the SNP6.0 chip which screens for 1.8 million markers of genetic variation. Secondly, we show that the accuracy of allele frequency estimates can be improved by using an ethnically similar reference sample data set for the PPC algorithm. Also presented is a novel pooling strategy which produces similar benefits to replicate pools.

## Results

### Detection rates

On the Affymetrix 500 k platform, the detection rate for the individually genotyped samples varied between 90% and 99% (mean: 96.9%, median: 97.6%, standard deviation: 2.3%). Samples which had lower detection rates than 90% were repeated. Pooled genotyping, yielded lower detection rates than individually genotyped samples of between 86% and 88%, likely due to the highly heterogeneous nature of the sample. These detection rates were nevertheless comparable with that published from other studies working on the Affymetrix 100 k and 500 k array sets. On the SNP6.0 platform, detection rates for pooled genotyping were between 96% and 98% (mean: 97.1%, median: 97.0%, standard deviation: 0.7%). As no prior-art exists for comparison, these high detection rates could be considered as par for pooled genotyping on the new platform.

### Validation of pooling strategy

**Figure 1:**
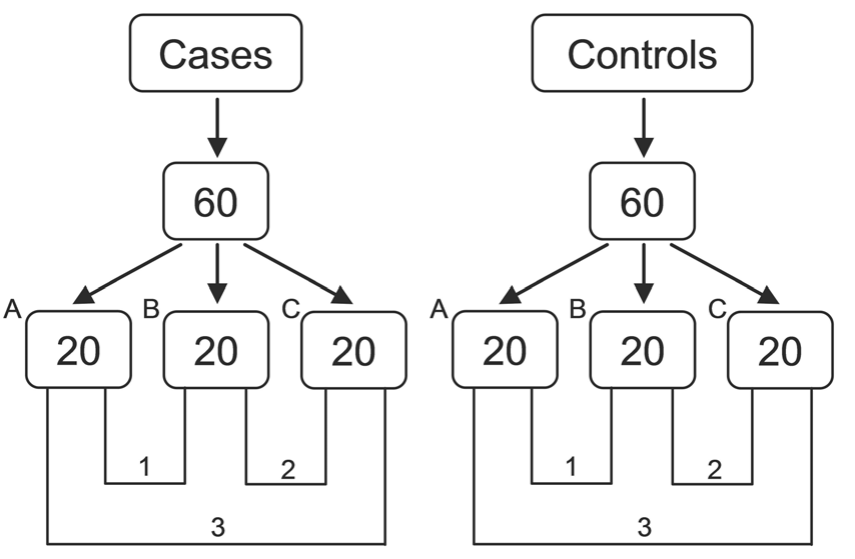
**Pooling strategy of overlapping sub-pools**.

To quantify the reliability of each of the three pools in the study groups (1, 2, and 3 in Figure 1), the allele frequency estimates obtained from the pools were compared with the actual allele frequencies for the 40 samples in each of the 3 pools. Estimated allele frequencies were calculated using beta values obtained from the individual genotyping. Subsequently, the allele frequencies from the 3 pools were averaged for each study group and were compared to the averaged allele frequencies of the 60 individually typed samples (Table 1).

**Table 1:** Comparing estimated and actual allele frequencies in sub-pools for 500 k platform.

**Actual AF compared with**:	Correlation	MAD error	(95% CI)
Case Pool 1	0.981	0.042	(0.0461-0.0463)
Case Pool 2	0.982	0.041	(0.0451-0.0453)
Case Pool 3	0.975	0.049	(0.0536-0.0540)
Average of Case Pools	0.987	0.035	(0.0389-0.0391)

Control Pool 1	0.977	0.044	(0.0496-0.0499)
Control Pool 2	0.976	0.049	(0.0526-0.0529)
Control Pool 3	0.978	0.046	(0.0501-0.0504)
Average of Control Pools	0.985	0.037	(0.0411-0.0414)

Actual allele frequencies obtained from individual genotyping of the 1^st ^set of 60 case and 60 control samples were compared with estimated allele frequencies from pooled genotyping of the same 120 samples. The comparison was carried out for samples in each of the sub-pools, and for averaged allele frequencies across the 3 sub-pools.

For pooled genotyping on the SNP6.0 platform, the known allele frequencies from the reference Sample Data Set were compared with the allele frequency estimates from each of the pool replicates. Subsequently, the estimated allele frequencies were averaged within each study group and were similarly compared with the known allele frequencies (Table 2).

**Table 2:** Comparing estimated and known allele frequencies in pool replicates for SNP6.0 platform.

**Known AF compared with**:	Correlation	MAD error	(95% CI)
Case Pool Replicate 1	0.983	0.042	(0.0435-0.0437)
Case Pool Replicate 2	0.986	0.038	(0.0397-0.0399)
Case Pool Replicate 3	0.988	0.036	(0.0373-0.0375)
Average of Case Pools	0.989	0.035	(0.0363-0.0365)

Control Pool Replicate 1	0.985	0.042	(0.0423-0.0425)
Control Pool Replicate 2	0.984	0.041	(0.0421-0.0423)
Control Pool Replicate 3	0.985	0.040	(0.0415-0.0417)
Average of Control Pools	0.988	0.036	(0.0372-0.0374)

Known allele frequencies obtained from the SNP6.0 Sample Data Set of the Hapmap CHB population were compared with estimated allele frequencies from pooled genotyping of 160 case and 160 control samples. The comparison was carried out for samples in each of the replicate-pools, and for averaged allele frequencies across the 3 replicates.

### Comparing different reference samples

Allele frequency estimates obtained by using beta values calculated from the reference 500 k Sample Data Set were compared with those estimates obtained using beta values calculated from samples individually genotyped in the lab (Table 3).

**Table 3:** Comparing accuracy of allele frequency estimates from different reference samples for 500 k platform (1).

Actual AF compared with:		Correlation	MAD error	(95% CI)
AF estimates from 500 k Sample Data Set	Cases	0.909	0.079	(0.1015-0.1020)
	Controls	0.907	0.079	(0.1024-0.1029)

AF estimates from individually typed samples	Cases	0.987	0.035	(0.0389-0.0391)
	Controls	0.985	0.037	(0.0411-0.0414)

Actual allele frequencies obtained from individual genotyping of the 1^st ^set of 60 case and 60 control samples were compared with estimated allele frequencies of the same set 120 samples. Estimated allele frequencies were separately calculated using beta values obtained from the 500 k Sample Data Set, and from the individually genotyped samples on the 500 k array.

There is a marked improvement in allele frequency estimates when they are calculated using beta values obtained from different reference samples. The averaged accuracy of the estimated allele frequencies (across cases and controls) improved from 90.8% to 98.6%, while the average error in the estimates improved by 0.06. To further confirm that such a difference exists when different reference samples are used to calculate beta values, allele frequencies were estimated for a second set of 60 cases and 60 controls pooled via the same strategy, using beta values calculated from the 500 k Sample Data Set and the individually typed samples (Table 4).

**Table 4:** Comparing accuracy of allele frequency estimates from different reference samples for 500 k platform (2).

Actual AF compared with:		Correlation	MAD error	(95% CI)
AF estimates from 500 k Sample Data Set	Cases	0.921	0.072	(0.0942-0.0946)
	Controls	0.914	0.077	(0.0998-0.1002)

AF estimates from individually typed samples	Cases	0.987	0.036	(0.0389-0.0392)
	Controls	0.985	0.039	(0.0422-0.0424)

Actual allele frequencies obtained from individual genotyping of the 1^st ^set of 60 case and 60 control samples were compared with the estimated allele frequencies of the 2^nd ^set of 60 case and 60 control samples. Estimated allele frequencies were separately calculated using beta values obtained from the 500 k Sample Data Set, and from the individually genotyped samples on the 500 k array.

Even in the second set of pooled samples, the average accuracy of allele frequency estimation seems to have improved by approximately 3% while error in allele frequency estimates improved by 0.05 when using the beta values obtained from in-house genotyped samples over those calculated from the reference sample set provided by Affymetrix.

For the pooled samples genotyped on the SNP6.0 platform, beta values calculated from the four Hapmap populations in the Sample Data Set were used to separately estimate different sets of allele frequencies. These estimates were compared with the known allele frequencies from the respective Hapmap populations (Table 5).

**Table 5:** Comparing accuracy of allele frequency estimates from different Hapmap reference samples for SNP6.0 platform.

Known AF compared with:		Correlation	MAD error	(95% CI)
AF estimated from CEU Sample Set (90)	Cases	0.889	0.097	(0.1095-0.1100)
	Controls	0.890	0.097	(0.1086-0.1090)

AF estimated from CHB Sample Set (45)	Cases	0.989	0.035	(0.0364-0.0365)
	Controls	0.988	0.037	(0.0372-0.0374)

AF estimated from JPT Sample Set (45)	Cases	0.984	0.040	(0.0426-0.0428)
	Controls	0.984	0.041	(0.0430-0.0432)

AF estimated from YRI Sample Set (90)	Cases	0.780	0.125	(0.1567-0.1574)
	Controls	0.782	0.124	(0.1550-0.1557)

AF estimated from All Sample Sets (270)	Cases	0.944	0.060	(0.0796-0.0799)
	Controls	0.945	0.059	(0.0789-0.0792)

Known allele frequencies from each of the four Hapmap populations (CEU, CHB, JPT and YRI) as provided in the SNP6.0 Sample Data Set, and an average allele frequency across the four populations were compared with the estimated allele frequencies of the 160 case and 160 control samples genotyped on the SNP6.0 platform. The estimated allele frequencies were calculated using beta values obtained from the four individual Hapmap populations and their collated genotypes in the same data set.

Estimated allele frequencies were most highly correlated with actual allele frequencies when the CHB Hapmap data set was used as a reference to calculate beta values with median absolute error of 0.036 (comparable to that obtained on the 500 k platform). When the CEU and YRI sample data sets were used to calculate allele frequencies from our pooled data, the accuracy of the allele frequency estimates were much lower (0.780 to 0.89). While the level of accuracy of estimation was higher when all the Hapmap samples were used (0.945, Table 5) for the SNP6.0 platform as compared to the 500 k Sample Data Set (0.908, Table 3), this could probably just be the due to the larger sample size (270 vs 48 in the latter).

### Validation of pooled genotyping

**Figure 2:**
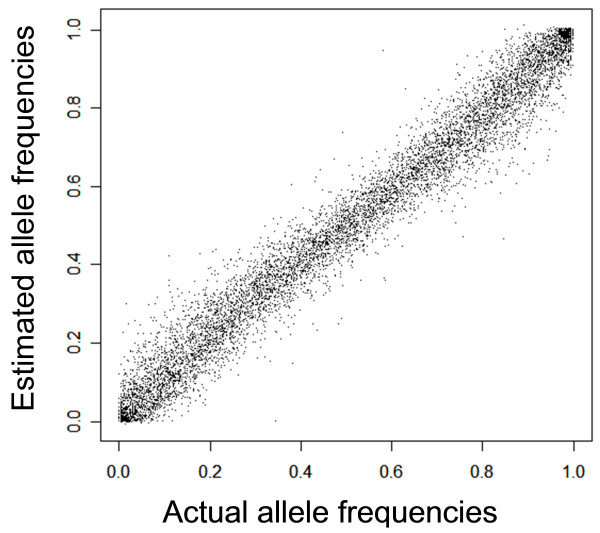
**Bi-plot comparing actual and estimated allele frequencies of a random selection of 10,000 SNPs**.

The results of pooled genotyping should only be considered valid if the allele frequency estimates generated from the pooled data is substantially confirmed by the average allele frequencies of all the individuals in the pooled samples. The actual and estimated allele frequencies of all the 500,568 SNPs in the Affymetrix 500 k array obtained from the individual and pooled genotyping of 120 samples were compared and were found to be highly correlated (Pearson's Correlation = 0.988). Figure 2 shows a visual comparison of the estimated and actual allele frequencies.

**Figure 3:**
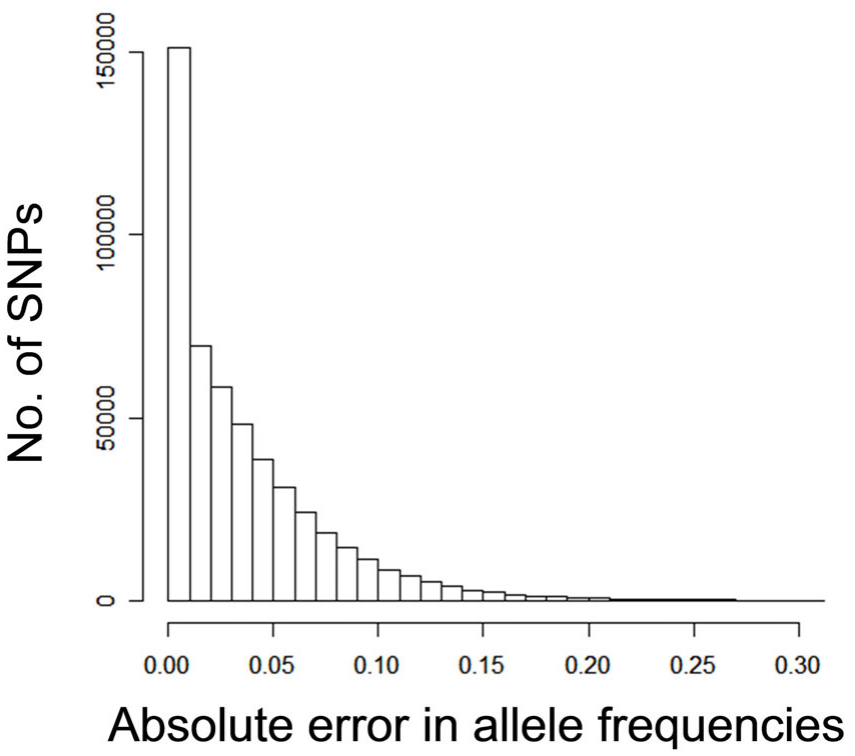
Frequency distribution of absolute errors in allele frequencies between individual and pooled genotyping

We calculated absolute errors in allele frequencies between the individual and pooled genotyping results, and looked at the distribution of these errors (Figure 3).

While the bi-plot comparison revealed that there were still allele frequency estimation errors of up to 0.2, studying the distribution of errors (Figure 3) showed that despite a high average error (mean = 0.036, median = 0.025, SD = 0.039), more than 92% (464,982) of the SNPs had their allele frequencies estimated to within 0.1 of the actual allele frequencies. When the data is fitted into a linear regression model, the regression coefficient (or slope of trend line) is 1.015, which further demonstrates the linear relationship between the actual and estimated allele frequencies.

### Factors affecting accuracy of allele frequency estimates

In individual genotyping, if a genotype cannot be assigned for a particular SNP for any particular sample, it is assigned as "NoCall". If a SNP is not called in the majority of samples genotyped, the allele frequency averaged across the remainder of the samples in which the SNP was properly called would not be representative of the whole sample population. As such, SNPs which had "NoCall" in too many samples could potentially be considered to have lower quality genotype calls. The genotype calling rates for all 500,568 SNPs were analysed across the 120 genotyped samples, and the SNPs were filtered based on number of samples with NoCall for each SNP (Table 6).

**Table 6:** Comparing errors in estimation of allele frequencies by filtering off uncalled SNPs.

NoCall cutoff	SNPs Analysed	Correlation	MAD error	95% CI
-	500568 (100%)	0.989	0.031	(0.0360-0.0362)
45	500488 (99.98%)	0.989	0.031	(0.0360-0.0362)
40	500353 (99.96%)	0.989	0.031	(0.0359-0.0362)
35	500036 (99.89%)	0.989	0.031	(0.0359-0.0361)
30	499222 (99.73%)	0.989	0.031	(0.0358-0.0360)
25	497469 (99.38%)	0.989	0.030	(0.0356-0.0358)
20	493792 (98.65%)	0.989	0.030	(0.0354-0.0356)
15	485709 (97.03%)	0.989	0.030	(0.0351-0.0353)
10	466758 (93.25%)	0.990	0.030	(0.0346-0.0348)
5	415562 (83.02%)	0.990	0.030	(0.0334-0.0337)
0	198749 (39.7%)	0.993	0.028	(0.0288-0.0291)

Actual allele frequencies obtained from individual genotyping were compared with estimated allele frequencies from pooled genotyping at various "NoCall" cutoffs.

In our data set of 120 samples, at most 45 were found with missing genotype calls for any SNP. While the majority of samples still had their genotypes called, the estimated allele frequencies were not too far off from the actual allele frequencies. The missing genotypes only started making a difference when SNPs with missing genotypes were excluded from the comparison of allele frequencies. When only SNPs which had 100% calls in all individuals were analyzed, correlation between estimated and actual allele frequencies improved marginally, with error improving by only 0.003. Generally, it was found that filtering of these SNPs which had seemingly lower quality showed no significant improvement in accuracy of genotype calls.

The complete list of SNPs was filtered based on different minor-allele frequency cutoffs to remove non-polymorphic and rare SNPs. Actual and estimated allele frequencies were compared and the results are listed in Table 7.

**Table 7:** Comparing errors in estimation of allele frequencies by filtering off rare SNPs.

MAF cutoff	SNPs Analysed	Correlation	MAD error	95% CI
1%	405478 (81%)	0.982	0.031	(0.0433-0.0435)
5%	355095 (70.94%)	0.976	0.032	(0.0449-0.0452)
10%	306552 (61.24%)	0.969	0.032	(0.0449-0.0452)
15%	260485 (52.04%)	0.961	0.031	(0.0439-0.0442)
20%	218147 (43.58%)	0.949	0.030	(0.0425-0.0428)

Actual allele frequencies obtained from individual genotyping were compared with estimated allele frequencies from pooled genotyping at various minor allele frequency cutoffs.

Our data showed that nearly 30% of the 500,568 SNPs screened were not really polymorphic in the study population. When only common SNPs were compared, the accuracy of allele frequency estimation decreased while average errors in allele frequencies increased by more than 0.03. While this might indicate that a large proportion of the "accuracy" we are observing is because of non-polymorphic SNPs in the study population, the correlation between actual and estimated allele frequencies still remained above 95%, which shows how accurate the estimates still are. While these results follow similar trends to those previously reported [5,12], our data shows less perturbations due to rare SNPs.

### Measuring reliability of allele frequency estimates

To further establish the performance of pooled genotyping, the sensitivity and specificity of the allele frequency estimates were tested across the range of minor-allele frequency cutoffs (Table 8). Sensitivity and specificity were calculated as follows:

**Table 8:** Sensitivity and specificity of estimated allele frequencies at various minor allele frequency cutoffs.

MAF cutoff	SNPs Analysed	Specificity		Sensitivity	
		
		Median	95% CI	Median	95% CI
0%	500568 (100%)	0.969	(0.9535-0.9538)	0.811	(0.4812-0.4886)
1%	405478 (81%)	0.958	(0.9438-0.9441)	0.832	(0.6226-0.6269)
5%	355095 (70.94%)	0.954	(0.9405-0.9408)	0.859	(0.7442-0.7464)
10%	306552 (61.24%)	0.952	(0.9388-0.9392)	0.879	(0.8030-0.8046)
15%	260485 (52.04%)	0.951	(0.9382-0.9386)	0.896	(0.8412-0.8425)
20%	218147 (43.58%)	0.951	(0.9383-0.9387)	0.909	(0.8676-0.8687)

Actual allele frequencies obtained from individual genotyping were compared with estimated allele frequencies from pooled genotyping. Sensitivity and specificity calculations were made at various minor allele frequency cutoffs.

As less common SNPs are increasingly excluded from the comparison, the accuracy of pooled genotyping in being able to correctly estimate allele frequencies which match the actual allele frequencies (specificity) reduced from 96.9% to 95.1%. This somewhat confirms the results observed in Table 7. Others have reported similar trends in accuracy of estimated allele frequencies of more common SNPs [18], but again our data still remains very reasonably accurate (specificity of 95.1%) even when considering 60% of the most common SNPs.

## Discussion

We hereby present a comprehensive genome-wide validation of pooled genotyping on the higher throughput SNP genotyping platforms. Using the complete Affymetrix 500 k array set as the basis of comparison, we have shown that the reliability and accuracy of pooled genotyping is as good as or improved over the previously tested 10 k and 100 k array sets. This comparison has been extended to the new SNP6.0 platform, which has yet been shown to be useful for pooled genotyping. We believe that this work would reaffirm that SNP-MaP is still a viable alternative to individually genotyping a large sample population.

### Novel Pooling strategy

Strategies for pooled genotyping have classically followed the path of having 3 identical replicate pools at the very least with the intent of "averaging" out the error normally associated with pooling [16,17,22]. The novel pooling strategy presented in this paper does not aim to replace the tried and tested method of replicates, but is rather proposed as an alternative. While it was performed in an attempt to evaluate the outcome of a thought experiment, the obtained results exceeded our expectations. Our pooling strategy involved the creation of 3 over-lapping pools from 3 sub-pools of 20 samples each. Comparing the accuracy of allele frequency estimates of each of the sub-pools to the average obtained across all 3 sub-pools (Table 1) showed that the novel pooling strategy of overlapping pools produced similar benefits in improved allele frequency estimation as compared to doing pooled replicates. As pooled replicates were not used for this part of our study, we chose to compare the capabilities of our overlapping sub-pools with that of pooled replicates as reported by others. The average correlation of estimated allele frequencies to actual allele frequencies improved by nearly 1% when the pools in each study group were considered as a whole and averaged. The average error in the allele frequency estimates was reduced by up to 0.01. These improvements in allele frequency estimates obtained from this novel pooling strategy compare well with those obtained from our replicate pools on the SNP6.0 platform as well as in other studies where even more chips were used [19]. While each of the samples was in effect replicated twice across 3 pools, they technically could not be considered as replicates. As such, the estimated allele frequencies from each of the 3 pools within our study groups were not as highly correlated with each other as they were with the actual allele frequencies they were estimating. Nonetheless, we showed that when the estimates obtained from the 3 pools were aggregated, they were able to more accurately estimate allele frequencies to a level comparably achieved by replicate pools [5,11,16]. This supports the fact that a sufficient number of replicates can control the pooling error to give results which can be very similar to those obtained from individual genotyping.

### Estimating Allele Frequencies

Individual genotyping classically produces genotype calls for each sample from which an average allele frequency can be calculated. However, in pooled genotyping, the microarray software is unable to assign a genotype due to the heterogeneous nature of the pooled sample, and the unequal hybridization to the various probes. As such, an algorithm to estimate allele frequencies from probe intensities was necessitated. To account for the unequal allelic amplification in pooled genotyping, relative allele signals (RAS) used together with a k-correction to improve accuracy of estimates was used initially [23]. This algorithm was extensively validated on the Affymetrix 10 k microarrays by various groups [9,11-15]. This relatively simple and yet accurate method of allele frequency estimation made it highly popular among researchers. So, even when a new algorithm (polynomial-based probe-specific correction or PPC) which improved on the highly popular RAS/k-correction method was proposed and was shown to give the best estimates of allele frequency from pooled genotyping on the Affymetrix 10 k platform [21], the tried and tested algorithm prevailed with its usefulness further extended to the Affymetrix 100 K microarray set [16], as well as the 500 K microarray set [5,19]. The main criticisms of the PPC algorithm were the time consuming computation in Perl and R, and the need for all 3 genotypes in the reference samples limiting the number of SNPs analysed [19]. Our group felt that with the rapid advancements in computing technology in recent years, the former criticism should not prevent usage of the more accurate PPC algorithm, even when considering the large volumes of data generated by the Affymetrix 500 k array set. The second criticism may not really be valid depending on the sample data set used to train the algorithm.

### Choice of Reference Data Set Affecting Accuracy of Allele Frequency Estimates

Regardless of the method used to estimate allele frequencies from the probe intensity data of pooled genotyping, the necessity of a set of reference samples is paramount. In most situations, allele frequency data from reference samples (usually from an appropriate Hapmap population) are used as a benchmark to compare the allele frequency estimates against. While the issue of reference samples was brought up [13] in the context of differential hybridization of heterozygous SNPs affecting accuracy of estimation of allele frequencies from pooled genotyping, no follow-up studies have been done in an attempt to quantify these differences. We have showed in this paper that the choice of reference samples does impact the accuracy of allele frequency estimates.

Our initial comparison of the accuracy of allele frequency estimates from pooled genotyping on the 500 k platform revealed that using a genetically homogeneous reference sample set, such as one from a particular ethnic group, produced estimated allele frequencies which were more accurate than using a more heterogeneous one. While our use of the same set of samples for individual and pooled genotyping provided a better indication of the capabilities of the 500 k platform in allelotyping, it might be thought that such a result would be expected given that the same samples were used for both. Our results from the first individual vs pool comparison (Table 3) were confirmed in the second comparison of actual and estimated allele frequencies (Table 4) from a completely different set of pooled samples, where we showed a similar high level of accuracy of the estimates.

This difference can be possibly attributed to availability of samples with all 3 genotypes for SNPs in the reference sample set. For the RAS method of calculating allele frequencies, the presence of heterozygous samples together with both homozygotes, allow the calculation of the k-correction which helps improve the accuracy of allele frequency estimates. Similarly, for PPC, the heterozygous samples allow the derivation of second-degree polynomials which increased accuracy of estimated allele frequencies by accounting for unequal hybridization efficiencies of different SNPs [21]. So a reference sample data set with a greater proportion of SNPs with heterozygous samples would, in theory, produce better allele frequency estimates than one with fewer SNPs with heterozygous samples. Furthermore, a genetically heterogeneous population should have more SNPs with heterozygous members. The 500 k Sample Data Set had 72.7% (364,140) of all SNPs with homozygous and heterozygous samples, while our individually typed samples had 63.3% or 316,623 SNPs with all 3 genotypes represented in the sample population. The difference in number of SNPs with all 3 genotypes between the two sample data sets reflects their heterogeneity; while the 500 k Sample Data Set was made up of representatives from the four major Hapmap populations, our own set of individually types samples were all ethnic Chinese. However, while this difference is expected given the ethnic differences in the two sample sets, the disparity in accuracy of allele frequency estimates produced by them is not. When our individually typed samples were used to estimated the polynomials (beta values) for PPC, the estimated allele frequencies were closer to the actual allele frequencies by more than 3% (mean difference in allele frequency of up to 0.05) when compared to the estimates obtained from the 500 k Sample Data Set (Table 3 and Table 4). These results indicate that a greater proportion of SNPs with 3 available genotypes in the reference sample set does not necessarily improve accuracy of allele frequency estimates. It could be that the SNPs which are or are not variable in the study population may not necessarily be the same as those in the reference population; as such variability (presence of homozygous and heterozygous samples for any particular SNP) of those SNPs in the reference population is not helpful in improving accuracy of estimated allele frequencies.

We believed that SNP variability was related to the ethnicity of the samples in the reference data set. While complete reference sample data sets from different ethnicities were not easily available for the 500 k platform, complete data for all 270 Hapmap samples was made available by Affymetrix when the SNP6.0 was released. This allowed us to compare the accuracy of allele frequencies from our pooled genotyping calculated using beta values from the four major Hapmap populations against the allele frequencies of those very populations. While we have yet to do individual genotyping on the SNP6.0 platform, such a comparison would still be valid as we have already shown that our Singapore Chinese samples are similar to the Hapmap Han Chinese (CHB) population (unpublished data). Our results (Table 5) confirmed our suspicions that the ethnicity of the reference data set is indeed important; higher levels of accuracy were observed when allele frequencies were estimated from beta values calculated using a reference population of similar ethnicity. While the accuracy of estimation improved when the four Hapmap populations were considered as a whole as compared to the 500 k Sample Data Set, this could have been due to the greater number of samples (270 vs 48) in the reference set. While the CEU and YRI data sets had significantly more informative SNPs with all 3 genotypes called (66.31% and 72.47% respectively), the CHB population data set still managed to produce better estimates of allele frequency with a relatively lower (55%) proportion of such SNPs. Neither the increased numbers in the CEU, YRI and combined data sets over the CHB or JPT reference data sets, nor the availability of heterozygous samples with both homozygotes improved the accuracy of allele frequency estimates. While we believe that the differences in accuracy of allele frequencies when using the different reference sample sets may be due to the rather disparate variability between the various Hapmap populations [24], the most important property of the reference sample set which would affect accuracy of allele frequency estimates is its ethnic background and whether it shared this with the study population.

The importance of a reference sample set which is genetically homogeneous with the study population in genome-wide association studies using pooled genotyping, might be taken to mean that if researchers are studying a population for which reference genotyping data is not available (most likely outside the 4 main Hapmap populations), they would need to perform a round individual genotyping so as to generate a set of reference data which they can use for subsequent pooling experiments. This greatly detracts from the benefits offered by pooled genotyping as a more economical and more efficient way of performing an initial whole genome scan as part of an association study. However, this is where genotyping repositories, as suggested by various authors [12,13], would come in useful, in providing complete reference data sets of populations not currently covered in the International Hapmap Project.

### Validation of Pooled Genotyping on High Throughput Platforms

In this paper, we reinforce the capabilities of SNP-MaP as an alternative to individual genotyping of hundreds or thousands of samples in a genome-wide case-control association study. While pooled genotyping had been previously validated on the smaller scale Affymetrix 10 k and 100 k array sets, similarly detailed analysis had not been done on the 500 k or newer SNP genotyping platforms. Previous validation studies have shown accuracies of pooled genotyping on the 10 k platform ranging from 0.923 [11] to 0.987 [13] and from 0.971 [16] to 0.983 [17] on the 100 k array set. While pooled genotyping seemed immensely popular using the relatively lower throughput 10 k and 100 k genotyping platforms, researchers did not seem equally enthused with the newer improved efficiency SNP genotyping chips [15]. This could have been due to the apprehension about the 'trade-offs' associated with trying to squeeze more probes onto a microchip. While both the 10 k and 100 k chips had 40 probes for each SNP, the 500 k and SNP6.0 arrays had it reduced to 24 and 6 per SNP respectively, with certain SNPs being represented by an extra 4 and 2 probes respectively.

Nonetheless, validation of pooled genotyping was indeed carried out on the 500 k arrays with estimation accuracies ranging from 0.926 [5] to 0.983 [19]. While Wilkening et al. used only 40% of the SNPs, (SNPs found on the Nsp I chip of the 500 k array set), Docherty et al. evaluated the performance of almost all the SNPs (> 90%) in the array set. Building on Docherty et al.'s work, we chose to base our study on the full repertoire of 500,568 SNPs. The high level of accuracy we have shown (Pearson's Correlation = 0.988) is comparable with that obtained by others. The estimated allele frequencies show minimal variability from the actual allele frequencies (mean error = 0.036), and is similarly comparable to previous studies. Despite the apprehension about pooled genotyping on the 500 k platform, we have shown that allelotyping of pooled samples on this platform is both reliable and accurate. These results add to the work done by others to further affirm that pooled genotyping is extremely viable on this higher throughput platform.

We took this analysis one step further by focusing on the currently available ultra high-throughput SNP genotyping SNP6.0 platform and the 906,600 SNPs it covered (the other 946,000 probes on the SNP6.0 chip were for the detection of copy number variations which are outside the scope of this paper). Estimated allele frequencies from our pooling experiment highly represented those from our selected reference data set (Pearson's Correlation = 0.989, mean error = 0.035). Despite the reduction in intensity data available per-SNP, the SNP6.0 platform seems equally well suited as its predecessors for SNP-MaP. Although our allele frequency estimates from pooled genotyping on the SNP6.0 platform were based on individual genotyping data of Hapmap CHB samples instead of the samples in the pools (which we used in our validation on the 500 k platform), we are still highly confident of its relevance due to the ethnic similarity of Hapmap CHB and our Singapore Chinese samples.

In the 10 k and 100 k arrays, relative allele signal data was readily available thus allowing the use of the RAS method to estimate allele frequencies together with the k-correction to account for unequal hybridization. While such data was directly unavailable for the 500 k data, various authors [12,15] provided scripts or formulae to extract this information from the raw intensity data. In the three generations of SNP chips, both PM (Perfect Match) and MM (Mis-Match) probes were present, thus allowing relative signal intensities to be calculated. However, with the newer SNP6.0 chip only PM probes were available, probably due to the increased coverage of genetic variants. With the availability of only PM signal intensities (instead of RAS signals), PPC was the only method for estimating allele frequencies from pooled genotyping data using only the PM probes while still accounting for unequal hybridization. Prior to this study, PPC had only been validated on the 10 k platform [19,21]. Following our validation of pooled genotyping on the 500 k array set using PPC for allele frequency estimation, the current ascertainment of the performance of the SNP6.0 array in SNP-MaP would be the first on such a high density microarray.

Previous studies [5,12,18] have suggested that high estimates of reliability of pooled genotyping are inflated by a variety of factors such as quality of genotype calls for certain SNPs, and rare or non-polymorphic SNPs. Both these factors were examined to evaluate their relationship with the accuracy of allele frequency estimates. We discovered (Table 6) that SNPs with missing genotype calls in the reference data set did not affect accuracy of estimated allele frequencies derived from beta values calculated from the reference samples unlike mentioned previously [12]. Excluding SNPs which were rare in the reference sample set (minor allele frequency < 5%) did cause accuracy of allele frequency estimates to reduce slightly to 0.976 (Table 7); however, this difference is minor, unlike what was reported before [5], and should not be taken as an indication that the high levels of accuracy observed were in fact due to non-polymorphic SNPs in the populations. As a measure of the performance of allele frequency estimation, sensitivity and specificity were calculated for subsets of SNPs following various minor allele frequency cut-offs. The high specificity (95.4%, Table 8) of allele frequency estimates of common SNPs (minor allele frequency > 5%) indicates that Type I errors in the approximation of true allele frequency are low while not really compromising on the sensitivity of the test (sensitivity = 85.9%).

Regardless of how we compared our pooled estimates of allele frequencies with the actual allele frequencies obtained from our individually typed samples and known allele frequencies from Hapmap CHB samples, the allele frequency estimates that we obtained proved to be extremely reliable. With reliability and validity improvements over that previously demonstrated on 10 k, 100 k and 500 k arrays, we have shown that both the 500 k and SNP6.0 platforms perform well in pooled genotyping.

While we have showed that pooled genotyping allows the estimation of allele frequencies which are highly accurate compared to the actual allele frequencies, it cannot be used to completely replace individual genotyping; the availability of actual genotype data as obtained from individual genotyping allows a more detailed analysis and understanding of the genomic variability in the sample population, and also permits linkage and haplotype analysis within the population. Furthermore, while the genotyping of pooled samples introduces errors, and the errors due to pooling are usually minimal, and random errors due to the array itself can be corrected for by having multiple pooled replicates [22], systematic errors due to the array itself might go unnoticed unless individual genotyping is done. Therefore, pooled genotyping would be best suited when relative instead of absolute allele frequencies are desired, such as in case control association studies. Even then, pooled genotyping should always be followed up by individual genotyping, such as in a two-stage study design [3], so as to validate the observations from the pooled estimates.

## Conclusions

In this study, we have successfully shown that pooled genotyping is a reliable and accurate method of performing a truly genome-wide scan by analysing the performance of the complete repertoire of SNPs on the Affymetrix 500 k high throughput genotyping platform. Using this comparison as the basis, we showed that SNP-MaP is highly viable on the latest even-higher throughput SNP6.0 platform. We believe that newer, even higher density SNP microarrays will be amenable to pooled genotyping following the strategy outlined. The accuracy of allele frequency estimates was shown to be improved by using the PPC algorithm and by using a reference population data set of similar ethnicity to the study population for the calculation of beta values for PPC. Lastly, a novel pooling strategy was explored, and was found to provide similar benefits as that observed in others' replicate pools.

## Methods

### Samples

The DNA samples used in this study were collected from ethnic Chinese participants as part of an on-going retrospective cross-sectional study on allergic diseases in Singapore (unpublished data) following standard protocols for informed consent. Approval to conduct the study was obtained from the National University of Singapore Institutional Review Board (NUS-IRB Reference Code: 07-023). Genomic DNA was extracted from buccal cells obtained from a mouthwash in 0.9% saline solution. In short, the buccal cells were pelleted and lysed; DNA was extracted using the phenol-chloroform phase-separation technique [25], purified by two washes in ethanol, with the DNA pellet resuspended in reduced Tris-EDTA buffer. Samples were quantified in triplicate on the Nanodrop (ND-1000). Samples which fell within a 1% error margin in the replicate measurements were subsequently diluted to 50 ng/μl, according to the requirements in the assay manual.

Samples were subsequently stratified into case and control groups according to their disease status as determined by ISAAC-derived questionnaires [26] and skin-prick test for common allergens in Singapore. As all the samples used were collected in the same sampling frame, they are all assumed to have the same level of genetic heterogeneity. A total of 560 case and control samples were used for the subsequent genotyping experiments. Firstly, 60 case and 60 control samples were selected for individual genotyping on the 500 k platform. These same 120 samples were also analysed in the first round of pooled genotyping. A separate set of 60 case and 60 control samples were used in the second round of pooling on the same platform. Lastly, 160 case and 160 control samples were used for the pooled genotyping on the SNP6.0 platform.

### Pooling

A novel pooling strategy, involving over-lapping sub-pools to form a larger pool, was used for pooled genotyping on the 500 k platform. Twenty samples from either the case or control group of samples were pooled in equal quantities of DNA to form a single sub-pool. This was repeated twice further to produce a total of 3 sub-pools (A, B and C in Figure 1) making up 60 samples in each of the study groups. Three pools of 40 samples (1, 2, and 3 in Figure 1) were created from these 3 sub-pools by merging them as follows: A+B, A+C, B+C. The 3 pools of 40 samples each were subsequently re-quantified on the Nanodrop to ensure accuracy of pooling prior to being genotyped. This pooling strategy was employed in the genotyping of the first two sets of 60 case and 60 control samples. In total, 240 samples were analysed by pooled genotyping on the 500 k platform.

For pooled genotyping on the SNP6.0 platform, 160 case and 160 control samples were pooled in equal quantities of DNA to form a single pool for each study group. The pooled samples were re-quantified to ensure accuracy of pooling prior to genotyping. A total of 320 samples were analysed by pooled genotyping on the SNP6.0 platform.

### Genotyping

For genotyping using the 500 k array set, individual and pooled DNA samples were similarly processed according to the protocol outlined in the GeneChip Mapping 500 k Assay Manual. The assay chips were washed and stained on the Fluidics Station 450, and were scanned on the GeneChip Scanner 3000 7G. Raw data was exported from the GeneChip Operating Software v1.4 (GCOS) for separate analysis of individual and pooled genotyping. In total, 120 chip pairs (NspI and StyI) were used for individual genotyping of 120 samples. Six chip pairs were used for pooled genotyping of the same 120 samples which were individually genotyped. Subsequently, 6 more chip pairs were used for pooled genotyping of the second set of 120 samples on the 500 k array platfrom. For the SNP6.0 arrays, while the protocol was similar, genotyping was outsourced to Origen Laboratories Pte Ltd in Singapore. Both the case and control pooled samples were genotyped in triplicate. In total, 6 chips were used for pooled genotyping of 320 samples on the SNP6.0 platform.

### Genotype calling for individually typed samples

The CEL files generated by GCOS were processed using the BRLMM algorithm as implemented in the Genotyping Console v2.1, and genotypes were called using the default settings. The exported genotypes, in the two-letter format (AA, AB and BB) were converted to allele frequencies of the A allele (1.0, 0.5 and 0 respectively) via a Perl script to simplify subsequent analysis in R [27].

### Reference Sample Data Sets

The Mapping 500 k Sample Data Set is made up of thirteen trios (5 HapMap CEPH trios, 5 HapMap Yoruban trios and three other non-HapMap trios) and 9 unrelated HapMap Asian samples [28]. The Genome-Wide Human SNP Array 6.0 Sample Data Set, on the other hand, is made up of all the 270 Hapmap samples consisting of 30 CEPH trios, 30 Yoruban trios, 45 unrelated Han Chinese samples and 45 unrelated Japanese samples [29]. These reference data sets are made up of raw probe intensities (.CEL files) and genotype calls (.CHP files).

### Allelotyping for pooled samples

The genotype call files together with the probe intensities of the individually typed samples were processed via Perl and R scripts to calculate the three polynomials (or beta values) required by the PPC algorithm. The 500 k and SNP6.0 Sample Data Sets obtained from Affymetrix were also similarly processed to calculate a separate set of beta values. Allele frequencies were subsequently estimated from the probe intensities of the pooled genotyping data following the PPC algorithm as outlined by Brohede *et al*., 2005 [21]. Separate allele frequency estimates were obtained using the beta values calculated from the individual genotyping and the different Sample Data Sets.

### Statistical Analysis

Pearson's correlation coefficient was calculated to determine "correlation" as a measure of accuracy between actual and estimated allele frequencies. The error in the estimation of allele frequencies was the absolute difference between actual and estimated allele frequencies. Median absolute deviation (MAD) was used as a more robust estimator of dispersion of errors than standard deviation or variance. A 95% confidence interval of the distribution of errors was used as an interval estimate of spread of estimation errors. All other statistical analyses and data manipulations were carried out in Microsoft Excel, Affymetrix Power Tools [30], R and via Perl scripts. The Perl and R scripts used in this study were modified from those published by Brohede *et al*., 2005, and are available in Additional file 1.

## Availability and Requirements

Operating system(s): Platform independent

Programming Language(s): Perl, R scripting

Licence(s): GNU General Public Licence

Restriction(s): No restrictions to use by non-academics.

## Authors' contributions

RA performed the individual and the majority of the pooled genotyping, carried out all the microarray data analysis, and drafted the manuscript. FTC conceived, designed and planned the study and edited the manuscript. Both authors have read and approved the final manuscript.

## Supplementary Material

Additional file 1 Script.zip containing the following Perl and R scripts
Scripts.txt – All R scripts, including usage of Perl scripts
call.p – Perl script to process genotype calls (500k data set)
extractfields.p – Perl script to extract range of columns from data file
extractprobes.pl – Perl script to process probe intensities (SNP6.0 data set)
field.p – Perl script to extract single column from data file
int.p – Perl script to process probe intensities (500k data set)
relint.pl – Perl script to process relative intensities (SNP6.0 data set)
sepprobe.pl – Perl script to separate probe intensities (SNP6.0 data set) Click here for file

## References

[B1] Brookes AJ The Essence of SNPs Gene 1999 234 177 186 10.1016/S0378-1119(99)00219-X 10395891

[B2] Hirschhorn JN Daly MJ Genome-Wide Association Studies For Common Diseases And Complex Traits Nature Reviews Genetics 2005 6 95 108 10.1038/nrg1521 15716906

[B3] Zuo Y Zou G Zhao H Two-Stage Designs in Case-Control Association Analysis Genetics 2006 173 1747 1760 10.1534/genetics.105.042648 16624925 PMC1526674

[B4] Wang WYS Barratt BJ Clayton DG Todd JA Genome-Wide Association Studies: Theoretical and Practical Concerns Nature Reviews Genetics 2005 6 109 118 10.1038/nrg1522 15716907

[B5] Docherty SJ Butcher LM Schalkwyk LC Plomin R Applicability of Dna Pools on 500 K Snp Microarrays for Cost-Effective Initial Screens in Genomewide Association Studies BMC Genomics 2007 8 10.1186/1471-2164-8-214 17610740 PMC1925094

[B6] Zaharieva I Georgieva L Nikolov I Kirov G Owen MJ O'Donovan MC Toncheva D Association Study in the 5q31-32 Linkage Region for Schizophrenia Using Pooled Dna Genotyping BMC Psychiatry 2008 8 10.1186/1471-244X-8-11 18298822 PMC2268687

[B7] Shifman S Johannesson M Bronstein M Chen SX Collier DA Craddock NJ Kendler KS Li T O'Donovan M O'Neill FA Genome-Wide Association Identifies a Common Variant in the Reelin Gene That Increases the Risk of Schizophrenia Only in Women PLoS Genetics 2008 4 e28 10.1371/journal.pgen.0040028 18282107 PMC2242812

[B8] Steer S Abkevich V Gutin A Cordell H Gendall K Merriman M Rodger R Rowley K Chapman P Gow P Genomic Dna Pooling for Whole-Genome Association Scans in Complex Disease: Empirical Demonstration of Efficacy in Rheumatoid Arthritis Genes and Immunity 2007 8 57 68 10.1038/sj.gene.6364359 17159887

[B9] Butcher L Meaburn E Dale P Sham P Schalkwyk L Craig I Plomin R Association Analysis of Mild Mental Impairment Using Dna Pooling to Screen 432 Brain-Expressed Single-Nucleotide Polymorphisms Molecular Psychiatry 2005 10 384 392 10.1038/sj.mp.4001589 15452586

[B10] Baum A Akula N Cabanero M Cardona I Corona W Klemens B Schulze T Cichon S Rietschel M Nothen M A Genome-Wide Association Study Implicates Diacylglycerol Kinase Eta (Dgkh) and Several Other Genes in the Etiology of Bipolar Disorder Molecular Psychiatry 2008 13 197 207 10.1038/sj.mp.4002012 17486107 PMC2527618

[B11] Butcher LM Meaburn E Liu L Fernandes C Hill L Al-Chalabi A Plomin R Schalkwyk L Craig IW Genotyping Pooled DNA on Microarrays: A Systematic Genome Screen of Thousands of SNPs in Large Samples to Detect QTLs for Complex Traits Behavior Genetics 2004 34 549 555 10.1023/B:BEGE.0000038493.26202.d3 15319578

[B12] Craig DW Huentelman MJ Hu-Lince D Zismann VL Kruer MC Lee AM Puffenberger EG Pearson JM Stephan DA Identification of Disease Causing Loci Using an Array-Based Genotyping Approach on Pooled DNA BMC Genomics 2005 6 10.1186/1471-2164-6-138 16197552 PMC1262713

[B13] Simpson CL Knight J Butcher LM Hansen VK Meaburn E Schalkwyk LC Craig IW Powell JF Sham PC Al-Chalabi A A Central Resource For Accurate Allele Frequency Estimation From Pooled DNA Genotyped On DNA Microarrays Nucleic Acids Research 2005 33 e25 10.1093/nar/gni028 15701753 PMC549427

[B14] Meaburn E Butcher LM Liu L Fernandes C Hansen V Al-Chalabi A Plomin R Craig I Schalkwyk LC Genotyping DNA Pools on Microarrays: Tackling the QTL Problem of Large Samples and Large Numbers of SNPs BMC Genomics 2005 6 10.1186/1471-2164-6-52 15811185 PMC1079828

[B15] Kirov G Nikolov I Georgieva L Moskvina V Owen MJ O'Donovan MC Pooled DNA Genotyping on Affymetrix SNP Genotyping Arrays BMC Genomics 2006 7 10.1186/1471-2164-7-27 16480507 PMC1382214

[B16] Meaburn E Butcher LM Schalkwyk LC Plomin R Genotyping Pooled DNA Using 100 K SNP Microarrays: A Step Towards Genomewide Association Scans Nucleic Acids Research 2006 34 e28 10.1093/nar/gnj027 16478714 PMC1368655

[B17] Macgregor S Visscher PM Montgomery G Analysis of Pooled DNA Samples on High Density Arrays Without Prior Knowledge of Differential Hybridization Rates Nucleic Acids Research 2006 34 e55 10.1093/nar/gkl136 16627870 PMC1440945

[B18] Yang H-C Lin C-H Hung S-I Fann CSJ A Comparison of Individual Genotyping and Pooled DNA Analysis for Polymorphism Validation Prior to Large-Scale Genetic Studies Annals of Human Genetics 2006 70 350 359 10.1111/j.1529-8817.2005.00232.x 16674557

[B19] Wilkening S Chen B Wirtenberger M Burwinkel B Försti A Hemminki K Canzian F Allelotyping of Pooled DNA with 250 K SNP Microarrays BMC Genomics 2007 8 10.1186/1471-2164-8-77 17367522 PMC1839100

[B20] Consortium TWTCC Genome-wide association study of 14,000 cases of seven common diseases and 3,000 shared controls Nature 2007 447 661 678 10.1038/nature05911 17554300 PMC2719288

[B21] Brohede J Dunne R McKay JD Hannan GN PPC: An Algorithm for Accurate Estimation of SNP Allele Frequencies in Small Equimolar Pools of DNA Using Data from High Density Microarrays Nucleic Acids Research 2005 33 e142 10.1093/nar/gni142 16199750 PMC1240117

[B22] Macgregor S Most Pooling Variation in Array-Based DNA Pooling is Attributable to Array Error Rather Than Pool Construction Error European Journal of Human Genetics 2007 15 501 504 10.1038/sj.ejhg.5201768 17264871

[B23] Hellard SL Ballereau SJ Visscher PM Torrance HS Pinson J Morris SW Thomson ML Semple CAM Muir WJ Blackwood DHR SNP Genotyping on Pooled DNAs: Comparison of Genotyping Technologies and a Semi Automated Method for Data Storage and Analysis Nucleic Acids Research 2002 30 e74 10.1093/nar/gnf070 12140336 PMC137092

[B24] Cheng Hu WJ Zhang W Wang C Zhang R Wang J Ma X Consortium TITDq Xiang K An Evaluation of the Performance Of Hapmap SNP Data in a Shanghai Chinese Population: Analyses of Allele Frequency, Linkage Disequilibrium Pattern and Tagging SNPs Transferability on Chromosome 1q21-q25 BMC Genetics 2008 9 18302794 10.1186/1471-2156-9-19 PMC2292209

[B25] Moore D Dowhan D Unit 2.1A: Purification and Concentration of DNA from Aqueous Solutions Current Protocols in Molecular Biology 2002 10.1002/0471142727.mb0201as59 18265306

[B26] Asher MI Keil U Anderson HR Beasley R Crane J Martinez F Mitchell EA Pearce N Sibbald B Stewart AW International study of asthma and allergies in childhood (ISAAC): rationale and methods European Respiratory Journal 1995 8 483 491 10.1183/09031936.95.08030483 7789502

[B27] Team RDC R: A Language and Environment for Statistical Computing: R Foundation for Statistical Computing 2008 http://www.R-project.org

[B28] Affymetrix Mapping 500 K Sample Data Set http://www.affymetrix.com/support/technical/sample_data/500k_data.affx

[B29] Affymetrix Genome-Wide Human SNP Array 6.0 Sample Data Set http://www.affymetrix.com/support/technical/sample_data/genomewide_snp6_data.affx 10.1186/1471-2164-14-506 PMC3727995 23885805

[B30] Affymetrix Affymetrix Power Tools http://www.affymetrix.com/partners_programs/programs/developer/tools/powertools.affx

